# Effect of microbiology comment nudging on antibiotic use in asymptomatic bacteriuria: A before-and-after quasi-experimental study

**DOI:** 10.1017/ice.2022.272

**Published:** 2023-09

**Authors:** Madeline G. Belk, Olivia D. Hammond, Callie C. Seales, Jonathan D. Edwards, Taylor D. Steuber

**Affiliations:** 1 Huntsville Hospital, Department of Pharmacy, Huntsville, Alabama; 2 TriStar Centennial Medical Center, Nashville, Tennessee; 3 Texas Tech University Health Sciences Center, Lubbock, Texas; 4 Auburn University Harrison School of Pharmacy, Pharmacy Practice, Huntsville, Alabama

## Abstract

**Objective:**

To describe the effect of a microbiology comment nudge on antibiotic use for asymptomatic bacteriuria (ASB).

**Design:**

Single-center, before-and-after, quasi-experimental study.

**Setting:**

Community-based, public, not-for-profit teaching hospital in the southeastern United States.

**Participants:**

Adult inpatients with a positive urine culture and the absence of urinary tract infection signs and symptoms.

**Intervention:**

Implementation of a microbiology comment nudge on urine cultures.

**Results:**

In total, 204 patients were included in the study. Antibiotics were less likely to be continued beyond 72 hours in the postimplementation group: 57 (55%) of 104 versus 38 (38%) of 100 (*P* = .016). They were less likely to have antibiotics continued beyond 48 hours: 60 (58%) of 104 versus 43 (43%) of 100 (*P* = .036). They were also less likely to have antibiotics prescribed at discharge 35 (34%) of 104 versus 20 (20%) of 100 (*P* = .028). In addition, they had fewer total antibiotic days of therapy: 4 (IQR, 1–6) versus 1 (IQR, 0–6) (*P* = .022).

**Conclusion:**

Microbiology comment nudging may contribute to less antibiotic utilization in patients with ASB.

Asymptomatic bacteriuria (ASB) is the isolation of 1 or more species of bacteria in a urine culture without signs or symptoms of a urinary tract infection (UTI). The Infectious Diseases Society of America (IDSA) recommends against the treatment of asymptomatic bacteriuria except in pregnancy and patients undergoing genitourinary procedures in which mucosal bleeding is expected.^1^ Approximately 20% of elderly, otherwise healthy females in the community and up to 80% of those hospitalized are affected.^2,3^ Inappropriate treatment of ASB is associated with emergence of resistant organisms and subsequent UTIs in females with a history of recurrent UTIs.^4^ Inappropriate treatment has been attributed to factors such as abnormal urinalysis results, older patients, and patients with altered mental status.^3,5^ Therefore, reduction in treatment of ASB is an important goal for antimicrobial stewardship programs (ASPs). Current strategies include clinical decision support to reduce urine testing, interactive education sessions, withholding culture results, and audit and feedback.^2,4,6–8^

Microbiology comment nudging refers to a guideline-based recommendation in which the recommendation is typed and reported on a culture and susceptibility report to guide antimicrobial therapy. Musgrave et al^9^ demonstrated this behavioral intervention to be effective in treatment of pneumonia. After implementation of a comment nudge in their study, de-escalation and discontinuation of unnecessary broad-spectrum antibiotics increased, reducing harm in patients hospitalized with pneumonia. Alternative microbiology reporting interventions, such as selective or modified reporting of cultures and susceptibilities, have also been shown to improve antibiotic prescribing patterns.^10^ This low-cost, simple behavioral intervention has the potential for high yield when incorporated into ASPs, including other types of infections.^9,10^

At our institution, we noticed a high tendency to treat asymptomatic patients with urine cultures growing <100,000 colony-forming units per milliliter (CFU/mL). Therefore, a comment nudge was initially approved for urine cultures with <100,000 CFU/mL, with the goal of reducing inappropriate treatment and a plan to expand to all urine cultures in the future. We evaluated the effect of implementing a microbiology comment nudge on treatment of positive urine cultures.

## Methods

### Study design

In this single-center, before-and-after, quasi-experimental study, we assessed the impact of implementing a microbiology comment nudge on antibiotic utilization in patients with ASB. The study was conducted at a 971-bed community teaching hospital in the southeastern United States. The comment nudge was manually entered by the microbiology laboratory staff into the medical records of patients growing <100,000 CFU/mL of bacteria at time of speciation. The comment stated, “Assess for urinary tract infection symptoms with urine cultures growing <100K CFU/mL. Antibiotic treatment is not recommended in patients with asymptomatic bacteriuria outside of pregnancy and urologic procedures.” The intervention was initiated in January 2021 after it was approved by the infectious diseases subcommittee of the pharmacy and therapeutics committee.

Pharmacists and physicians were offered education via an online presentation which included education regarding ASB, when to treat ASB, and information regarding the microbiology comment. This presentation was followed by a survey to assess comprehension of the material prior to implementation. Patients admitted between February 2020 and December 2020 served as the preintervention control group, and patients admitted between February 2021 and September 2021 comprised the postintervention group. The study was approved by the respective institutional review boards and committees with waivers of consent.

### Inclusion and exclusion criteria

Patients were included who were aged ≥18 years and were admitted to the hospital with positive urine cultures growing <100,000 CFU/mL of bacteria without documented symptoms of a UTI including dysuria, urinary frequency or urgency, costovertebral angle tenderness, or systemic signs of infection, including fever or hemodynamic instability. Patients were excluded if they were undergoing a planned urologic procedure or were pregnant. Patients were also excluded if they were receiving antibiotics for another indication, were not treated as an inpatient, or had cultures growing mixed flora or yeast alone. Repeat or duplicate cultures were also excluded.

### Data collection, definitions, and outcomes

Patients were screened if they had a positive urine culture with <100,000 CFU/mL bacteria. Data were collected by manual chart review and symptoms (or lack thereof) were identified based on electronic documentation. Baseline and demographic characteristics, comorbidities, hospitalization characteristics, and urinalysis and urine culture results were collected. The primary end point was antibiotic treatment of ASB for >72 hours after initiation. Secondary end points included antibiotics initiated, antibiotic treatment for >48 hours, antibiotics prescribed at discharge, and total antibiotic days of therapy (DOT). Exploratory end points included hospital length of stay (LOS), 30-day readmission, and 30-day mortality.

### Statistical analysis

A sample size of 194 patients was estimated to detect a 20% reduction in the primary outcome, with a power of 80% and an α of 0.05 for significance. Descriptive and inferential statistics were used to analyze data. Bivariate analyses were conducted for the study population; the Pearson χ^2^ or the Fischer exact test was used to compare categorical variables, and the Student *t* test or the Mann-Whitney *U* test was used to compare continuous variables, as appropriate. Logistic regression analysis was conducted to identify variables associated with antibiotic discontinuation by 72 hours while controlling for confounders. Variables with potential to be associated with these outcomes in bivariate analysis (*P* < .20) and with clinical rationale were entered into the regression model and were removed stepwise using backward elimination. Model fit was assessed using the Hosmer-Lemeshow goodness-of-fit test with nonsignificant results considered adequate. An adjusted odds ratio in the final model with a confidence interval not including 1.0 was considered statistically significant. All statistical tests were two-sided, and *P* < .05 was considered statistically significant. Statistical analyses were performed using SPSS version 27.0 software (IBM, Armonk, NY).

## Results

**Figure 1.**
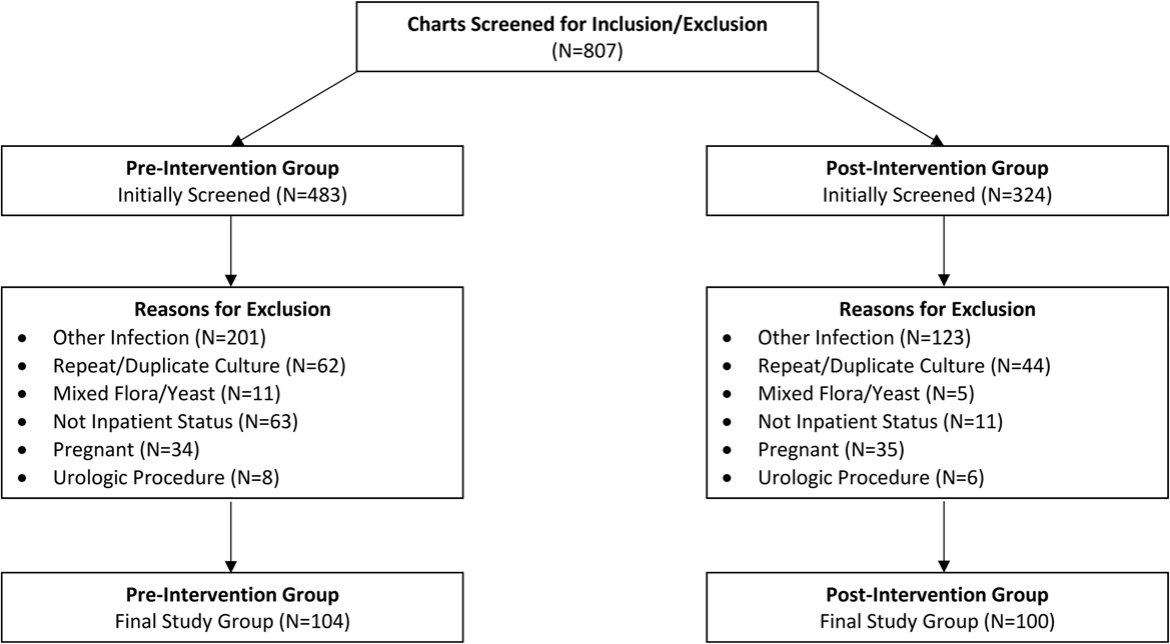
Patients Meeting Inclusion and Exclusion Criteria

In total, 807 encounters were initially screened, and 204 were included in the final analysis: 104 patients in the preintervention group and 100 patients in the postintervention group (Fig. 1). Baseline characteristics and demographics were similar between groups (Table 1). Patients were ∼72 years old, most were female, and the median Charlson comorbidity index (CCI) score was 4. Most patients were positive for leukocyte esterase. The most commonly identified organism was *Escherichia coli*, which accounted for roughly one-third of isolates, followed by *Enterococcus* spp, *Klebsiella* spp, and *Proteus* spp. These results are displayed in Table 2.

**Table 1.** Baseline Demographics of the Study Population

Characteristic	Preintervention Group (n = 104), No. (%)^a^	Postintervention Group (n = 100), No. (%)^a^
**Demographics and characteristics**		
Age, mean y (IQR)	76 (66–82)	72 (63–80)
Sex, female	66 (63)	72 (72)
β-lactam allergy	24 (23)	23 (23)
CCI, mean (IQR)	4 (3–5)	4 (3–5)
WBC >12,000/mm^3^ or <4,000/mm^3^	26 (25)	22 (22)
Temperature >38°C or <36°C	4 (4)	4 (4)
Altered mental status documented	15 (14)	21 (21)
Infectious disease consultation	15 (14)	12 (12)
**Urinalysis characteristics**		
Nitrite positive	14 (13)	9 (9)
Leukocyte esterase positive	87 (84)	86 (86)
Pyuria (>10 WBC/mm^3^)	64 (62)	65 (65)
**Urine culture characteristics**		
Multidrug-resistant organism	15 (14)	20 (20)
Extended-spectrum β-lactamase	4 (4)	4 (4)
>1 organism identified	11 (11)	12 (12)

Note. IQR, interquartile range; CCI, Charlson comorbidity index; WBC, white blood cells.

a Units unless otherwise specified.

**Table 2.** Species of Bacteria Identified in the Study Population

Bacterial Species	Preintervention Group (n = 104), No. (%)	Postintervention Group (n = 100), No. (%)
*Escherichia coli*	31 (30)	33 (33)
*Klebsiella* spp	13 (13)	11 (11)
*Proteus* spp	11 (11)	12 (12)
*Staphylococcus aureus*	4 (4)	2 (2)
Coagulase-negative *Staphylococcus*	3 (3)	5 (5)
*Enterococcus* spp	26 (25)	32 (32)
*Enterobacter* spp	8 (8)	2 (2)
*Citrobacter* spp	2 (2)	2 (2)
*Acinetobacter baumannii*	1 (1)	0 (0)
*Morganella morganii*	2 (2)	2 (2)
*Pseudomonas aeruginosa*	14 (13	9 (9)
*Serratia marcescens*	0 (0)	2 (2)

Primary and secondary end points are displayed in Table 3. The primary end point of antibiotic therapy for ASB for >72 hours occurred more frequently in the preintervention group compared to the postintervention group: 57 (55%) versus 38 (38%; *P* = .016). Additionally, antibiotic therapy for >48 hours occurred less frequently in the postintervention group. Antibiotics prescribed at discharge and total antibiotic DOT were also reduced. There was no difference in the incidence of antibiotics being initiated for ASB or exploratory outcomes, except for hospital LOS, which was longer in the postintervention group.

**Table 3.** Primary and Secondary End Points of the Study Population

End Point	Preintervention Group (n = 104), No. (%)^a^	Postintervention Group (n = 100), No. (%)^a^	*P* Value
**Primary end point**			
Antibiotics continued beyond 72 h^b^	57 (55)	38 (38)	.016
**Secondary end points**			
Antibiotics continued beyond 48 h^b^	60 (58)	43 (43)	.036
Antibiotics initiated	66 (63)	54 (54)	.17
Antibiotics prescribed at discharge	35 (34)	20 (20)	.028
Antibiotic DOT (median, IQR)	4 (0–7)	1 (0, 6)	.022
**Exploratory end points**			
Length of stay, median d (IQR)	5 (3–8)	6 (4–9)	.013
30-d readmission	13 (13)	15 (15)	.604
30-d mortality	2 (2)	4 (4)	.380

Note. DOT, days of therapy; IQR, interquartile range.

a Units unless otherwise specified.

b From time antibiotics were initiated.

Table 4 lists the significant results of the logistic regression analysis. Notably, postcomment nudge implementation was associated with increased likelihood of antibiotic discontinuation for ASB at 72 hours after initiation (adjusted odds ratio, 2.5; 95% confidence interval, 1.3—4.9; *P* = .006). Cultures growing >1 organism and patients with CCI scores <4 were also significant predictors of antibiotic discontinuation, whereas patients with pyuria had a decreased likelihood of discontinuation.

**Table 4.** Logistic Regression Analysis of Characteristics Associated with Antibiotic Discontinuation by 72 Hours

Variable^a^	OR (95% CI)	Adj OR (95% CI)	*P* Value
After comment nudge implementation	2.7 (1.3–5.3)	2.5 (1.3–4.9)	.006
>1 organism Identified	2.6 (0.9–9.4)	3.4 (1.1–10.4)	.035
CCI <4	4.2 (1.7–10.2)	4.0 (1.7–9.6)	.002
Pyuria	0.5 (0.3–1.1)	0.5 (0.2–0.9)	.041

Note. OR, odds ratio; Adj OR, adjusted odds ratio; CI, confidence interval; CCI, Charlson comorbidity index.

a Additional variables included in initial model that were nonsignificant: age<75 years, sex, various bacterial species, multidrug-resistant organism, infectious disease consult ordered, and altered mental status.

## Discussion

Antibiotic resistance is a growing public health threat and is strongly influenced by extended durations of antibiotics or inappropriate utilization.^4,11^ A commonly overtreated indication is ASB, despite IDSA guidance.^1^ The incidence of inappropriate treatment of ASB as defined by the IDSA was as high as 83% of hospitalized patients in a cohort study of almost 3,000 patients.^3^ Furthermore, in a study that included all hospitalized patients with urine culture results of >1,000 CFU/mL without documented symptoms, the treatment rate was 38%.^5^ This finding highlights significant opportunities for ASPs to reduce unnecessary treatment. Additionally, repercussions to inappropriate treatment include increased risks of antimicrobial resistance, *Clostridioides difficile* infection, and development of UTI shortly after therapy.^1^ Previous studies have found that the inappropriate treatment of ASB may lead to more subsequent symptomatic UTI, and to increased likelihood of adverse drug reactions.^12,13^ All of these important considerations warrant stewardship interventions to prevent the unnecessary treatment of ASB.

Our findings suggest that implementation of the simple behavioral intervention of a microbiology comment nudge reduced 4 metrics: (1) antibiotic continuation beyond 72 hours, (2) antibiotics continued beyond 48 hours, (3) antibiotics prescribed at discharge, and (4) total antibiotic DOT. Although the absolute reductions in these end points was relatively small, implementing a comment nudge is a very low-cost, low-resource intervention. Our findings of reduced antibiotic utilization from a comment nudge echoes findings from similar studies. Specifically, a study by Daley et al^14^ evaluated withholding culture and susceptibility results and replacing the results with, “This POSITIVE urine culture may represent asymptomatic bacteriuria or urinary tract infection. If urinary tract infection is suspected clinically, please call the microbiology laboratory … for identification and susceptibility results” in patients with positive urine cultures. Furthermore, appropriate treatment increased with this intervention (80% vs 52.7%; *P* = .002).^14^ However, these researchers included patients with symptomatic UTI as well, whereas we evaluated specifically asymptomatic patients. Additionally, Leis et al^7^ demonstrated that withholding positive urine culture results in noncatheterized inpatients led to reduced antimicrobial therapy (48% vs 12%; *P* = .002) when the following microbiology comment was added: “The majority of positive urine cultures from inpatients without an indwelling urinary catheter represent asymptomatic bacteriuria. If you strongly suspect that your patient has developed a urinary tract infection, please call the microbiology laboratory.” Comparatively, we found a more modest decrease in antibiotic utilization than the aforementioned studies, which is likely explained by those studies involving withholding culture and susceptibility results unless providers contacted the microbiology department.^7,14^ Our results demonstrate that comment nudging may independently reduce antibiotic utilization in ASB without involving an extra step, albeit to a lesser extent.

We did not detect a difference in antibiotics initiated for asymptomatic urine cultures. However, this finding can possibly be explained by some providers ordering antibiotics when abnormalities in the urinalysis are seen prior to culture results with the comment nudge returning. We did not detect a difference in our exploratory end points of 30-day readmission or mortality, but we did see an increase in LOS in the postintervention group. Another study reported a significant reduction in LOS after implementing a comment nudge.^14^ These conflicting findings demonstrate that this finding may be due to confounding variables we did not account for and could be a topic for future investigation.

We also evaluated variables associated with continued antibiotic treatment beyond 72 hours. Prescribers were more likely to discontinue antibiotics by 72 hours after implementation of the comment nudge, highlighting the effectiveness of this intervention. Furthermore, discontinuation of antibiotics was more likely to occur if the urine culture had >1 organism. This finding could possibly be explained by providers being more convinced of contamination with additional organisms present. Finally, discontinuation was more likely to occur if the patient’s CCI score was <4. This finding can likely be explained by the perception that patients with more comorbidities or who appear “sicker” should be treated. Pyuria was shown to negatively predict discontinuation of antibiotics by 72 hours. This finding is in line with other studies.^3,5^ Additional variables not accounted for may have influenced these findings, such as indwelling urinary catheters.

This study had several notable strengths and limitations. The study included enough patients in the sample size to meet statistical power. To our knowledge, this is the largest study evaluating a solution to the inappropriate treatment of ASB. Additionally, the groups were evenly matched with similar baseline characteristics. This study also had several limitations. The retrospective design limited internal validation. Additionally, the design provided barriers due to data mining, unmeasured confounders, and chart review, which may not have adequately captured presence or absence of urinary tract symptoms. Also, significant bias in assessment may have resulted from incomplete documentation. Additionally, the presence of UTI symptoms may have been difficult to detect in some elderly, frail inpatients due to the potential inability to give a reliable history, making the true diagnosis of ASB difficult to achieve. However, IDSA guidelines for ASB recommend against treatment in this population in the absence of local genitourinary symptoms or other systemic signs of infection even though diagnosis is difficult to achieve.^1^ Additionally, some patients may have completed a full course of antibiotics by the 72-hour mark; however, other end points indicate overall reduced antibiotic utilization. Furthermore, given the design of the study and data-mining process, our exclusion rate was high. However, most exclusions were made due to the presence of another infection that was being treated or documented UTI or systemic symptoms and helped identify a population that would potentially benefit from a comment nudge. Also, we did not correct for multiple comparisons, which could have led to a type 1 error. However, multiple findings were significant, so it is less likely due to chance alone. Another limitation was the fact that we did not go by the IDSA definition of ASB and only included the comment nudge on urine cultures with <100,000 CFU/mL of bacteria. This parameter was decided on by the infectious diseases subcommittee as an initial starting point to accommodate providers and because we had observed a high treatment rate for this patient population. Based on the data from this study, the comment nudge is now applied to all urine cultures regardless of CFU count. Application to all cultures is an opportunity for future investigation. Other potential confounders include the inability to educate providers in person about the comment nudge due to COVID-19 distancing guidelines. However, an online educational presentation was made available and education occurred in other ways, such as during inpatient medicine rounds or during Antibiotic Awareness Week. Future educational opportunities could be employed to continue to support the intervention, although these alone may have confounded results.

In conclusion, microbiology comment nudging may reduce the number of patients treated for ASB and contribute to reduced antibiotic utilization.

## References

[1] Nicolle LE , Gupta K , Bradley SF , Colgan R , DeMuri GP , Drekonja D , et al. Clinical practice guideline for the management of asymptomatic bacteriuria: 2019 update by the Infectious Diseases Society of America. Clin Infect Dis 2019;68:e83–e110. 30895288 10.1093/cid/ciy1121

[2] Nicolle LE. Asymptomatic bacteriuria. Curr Opin Infect Dis 2014;27:90–96. 24275697 10.1097/QCO.0000000000000019

[3] Petty LA , Vaughn VM , Flanders SA. Risk factors and outcomes associated with treatment of asymptomatic bacteriuria in hospitalized patients. JAMA Intern Med 2019;179:1519–1527. 31449295 10.1001/jamainternmed.2019.2871 PMC6714039

[4] Keller SC , Feldman L , Smith J , Pahwa A , Cosgrove SE , Chida N. The use of clinical decision support in reducing diagnosis of and treatment of asymptomatic bacteriuria. J Hosp Med 2018;13:392–395. 29856886 10.12788/jhm.2892 PMC6329386

[5] Grein JD , Kahn KL , Eells SJ , et al. Treatment for positive urine cultures in hospitalized adults: a three medical center survey of prevalence and risk factors. Infect Control Hosp Epidemiol 2016;37:319–326. 26607408 10.1017/ice.2015.281 PMC5089900

[6] Osiemo D , Schroeder DK , Klepser DG , Van Schooneveld TC , Watkins AB , Bergman SJ. Treatment of asymptomatic bacteriuria after implementation of an inpatient urine culture algorithm in the electronic medical record. Pharmacy (Basel) 2021;9:138. 34449690 10.3390/pharmacy9030138 PMC8396163

[7] Leis JA , Rebick GW , Daneman N , et al. Reducing antimicrobial therapy for asymptomatic bacteruria among noncatheterized inpatients: a proof-of-concept study. Clin Infect Dis 2014;58:980–983. 24577290 10.1093/cid/ciu010

[8] Daniel M , Keller S , Mozafarihashjin M , Pahwa A , Soong C. An implementation guide to reducing overtreatment of asymptomatic bacteruria. JAMA Int Med 2018;178:271–276. 10.1001/jamainternmed.2017.7290 29228072

[9] Musgrove MA , Kenney RM , Kendall RE , et al. Microbiology comment nudge improves pneumonia prescribing. Open Forum Infect Dis 2018;5:ofy162. 30057928 10.1093/ofid/ofy162 PMC6057519

[10] Langford BA , Leung E , Haj R , et al. Nudging in microbiology laboratory evaluation (NIMBLE): a scoping review. Infect Control Hosp Epidemiol 2019;40:1400–1406. 31679535 10.1017/ice.2019.293

[11] Teshome BF , Vouri SM , Hampton N , Kollef MH , Micek ST. Duration of exposure to antipseudomonal β-lactam antibiotics in the critically ill and development of new resistance. Pharmacother 2019;39:261–270. 10.1002/phar.2201 PMC6507412 30506852

[12] Cai T , Mazzoli S , Mondaini N , et al. The role of asympomatic bacteriuria in young women with recurrent urinary tract infections: to treat or not to treat? Clin Infect Dis 2012;55:771–777. 22677710 10.1093/cid/cis534

[13] Trestioreanu AZ , Lador A , Sauerbrun-Cutler MT , Leibovici. Antibiotics for asympomatic bacteriuria. Cochrane Database Syst Rev 2015;4:CD009534. 25851268 10.1002/14651858.CD009534.pub2 PMC8407041

[14] Daley P , Garcia D , Inayatullah R , Penney C , Boyd S. Modified reporting of positive urine cultures to reduce inappropriate treatment of asymptomatic bacteriuria among nonpregnant, noncatheterized inpatients: a randomized controlled trial. Infect Control Hosp Epidemiol 2018;39:814–819. 29804552 10.1017/ice.2018.100

